# Efficacy, safety and pharmacokinetics of clofarabine in Chinese pediatric patients with refractory or relapsed acute lymphoblastic leukemia: a phase II, multi-center study

**DOI:** 10.1038/bcj.2016.8

**Published:** 2016-02-26

**Authors:** A Lu, Y Fang, X Du, Y Li, Z Cai, K Yu, L Zhao, B Wang, J Wu, Y Cheng, Y Zuo, Y Jia, F Tan, L Ding, J Lu, L Zhang, X Huang

**Affiliations:** 1 Department of Pediatrics, Peking University People's Hospital, Beijing, China; 2 Department of Phase 1 Clinical Trial, Peking University People's Hospital, Beijing, China; 3 Guangdong General Hospital, Guangdong Academy of Medical Sciences, Guangzhou, China; 4 The First Hospital of China Medical University, Shenyang, China; 5 The First Affiliated Hospital, Zhejiang University, Hangzhou, China; 6 The First Affiliated Hospital of Wenzhou Medical University, Wenzhou, China; 7 Betta Pharmaceuticals Co., Ltd, Hangzhou, China; 8 Institute of Hematology, Peking University People's Hospital, Beijing, China

Acute lymphoblastic leukemia (ALL) is the most common malignancy in children and adolescents, accounting for ~75% of all childhood leukemia.^1^ Despite the advancement in treating newly diagnosed ALL, patients with refractory or relapsed (R/R) disease have poor outcome, and combinational chemotherapies induce complete response in about 35–42% of the children under this condition.^2^ Thus, new antileukemic agents are urgently needed.

Clofarabine is a second-generation purine nucleoside analog, which requires intracellular phosphorylation by deoxycytidine kinase to be metabolized to the triphosphate form necessary for cytotoxic effect.^3^ It has been approved for treating pediatric R/R ALL patients based on several phase II studies, with a response rate of ~26–30% in the Western population.^4, 5^ The most common adverse events (AEs) reported in phase I trial were transient liver dysfunction, skin rashes, palmoplantar erythrodysesthesia and mucositis.^6^ Despite its antileukemic activities and acceptable toxicity in the Western population,^7, 8, 9, 10^ little data are available on Asian patients with R/R ALL. Therefore, we performed this multi-center, phase II study in China to evaluate the activity, safety and pharmacokinetics of clofarabine monotherapy in pediatric patients with R/R ALL.

Eligible patients were pediatric patients (younger than 21 years old) with R/R ALL confirmed by histology. Relapsed ALL was defined as ALL that relapsed after at least two times of antileukemia therapy. Normal cardiac, hepatic and renal function and Eastern Cooperative Oncology Group performance status (0–2) were also needed. Patients with AEs who had not recovered from prior therapy within 3 months after allogeneic or autologous stem cell transplantation and with central nervous involvement or active infection were excluded. The detailed inclusion and exclusion criteria are listed in Supplementary Information Method section.

The primary end point was overall response rate (ORR), comprised of complete remission (CR) and CR with incomplete recovery of courts (CRi), according to the NCCN guidelines for ALL. This trial was reviewed by the institutional ethics committees and was conducted according to the Declaration of Helsinki. Written informed consent was obtained from each patient or their legal guardians. This study was registered as NCT02544789.

Clofarabine was administered intravenously at 52 mg/m^2^ over 2 h daily for five consecutive days. During the first two induction cycles, patients without objective response were taken off the study, and responsive patients continued to receive consolidation for a maximum 11 cycles if their non-hematological toxicity were below grade 3. A 25% dose reduction was required when grade 3 or higher non-hematological, non-infectious events occurred and recovered within 14 days. The response was determined using the NCCN guidelines for ALL. The methods for response assessment are detailed in the Supplementary Information Method section. AEs were graded using the National Cancer Institute (Rockville, MD, USA) Common Terminology Criteria for AE Version 3.0 (NCICTC-AE 3.0).

Four patients signed written informed consent to blood draws for pharmacokinetic analysis (Supplementary Information Method and Supplementary Figure 1). The data were analyzed by the compartmental model using DAS 2.1.1 software (BioGuider Co., Shanghai, China) to assess *C*_max_ (peak plasma concentration), AUC (area under the plasma concentration−time curve), *T*_max_ (time to achieve *C*_max_), serum clearance, half-life time and other parameters.

Fisher's test was used for the comparison of response rate. Kaplan−Meier methodology was used to estimate time-to-event outcomes. All analyses were carried out with SAS statistical software (SAS Institute Inc., Cary, NC, USA), version 9.1.3, and *P<*0.05 was considered statistically significant.

Forty-four patients were enrolled between 30 June 2009 and 25 November 2011 from five centers in China. Table 1 lists the characteristics of these patients. The median age was 13 years (range, 5–21 years), where most patients were male (76.7%) and with B-cell phenotype disease (76.7%). The median number of prior chemotherapies was three, and four patients had one prior hematopoietic stem cell transplantation. Seventeen (39.5%) patients were refractory to the last therapeutic regimen. The number of cycles the patients in this study received ranged from 1 to 11, with a median of two cycles. The median dose received was 430 (138.6, 990.0) mg.

Forty-three patients were evaluable for efficacy. Two achieved CR, and 17 achieved CRi, resulting in an ORR of 44.2% (95% CI 29.1%, 60.1%) with a median duration of 14.6 weeks (95% CI 14.0, 25.4 weeks, Figure 1). Better responses were seen in younger patients (younger than 14 years, ORR 60.9%, 14/23) and in patients with B-cell lineage disease (ORR 57.6%, 19/33). Other clinical features showed no influence on ORR. Responses are summarized in Supplementary Table 2. The median survival was 40.6 weeks (95% CI 21.0, 178.0) for all patients (Figure 1). Seven patients proceeded to hematopoietic stem cell transplantation after clofarabine treatment, in which five were alive at the cutoff time with an estimated median survival time of 92 weeks.

All 44 patients experienced at least one AE. The most common treatment-related AEs were neutropenia (88.6%), thrombocytopenia (88.6%), anemia (86.4%), nausea (45.5%), vomiting (36.4%), increased alanine aminotransferase (34.1%) and febrile neutropenia (29.6%). Supplementary Table 3 lists all grades of treatment-related AEs reported in ⩾5% of the patients. More than 85% of patients had grade 3 or higher hematologic AEs. The most common grade 3 or higher non-hematological AEs were hepatic toxicity and skin disorders. Four patients (9.1%) experienced severe AEs, in which two elevated transaminases were treatment related. The details of all severe AEs are summarized in Supplementary Information. Dose reduction was seen in one patient due to grade 4 hematologic toxicity in the first treatment cycle; clofarabine was reduced from 62.4 to 45 mg in the second cycle; thereafter no grade 3 or higher AEs were seen.

Supplementary Figure 1 lists the level of 24-h plasma clofarabine after the initial infusion. The peak level of plasma clofarabine occurred at the end of the infusion. Clofarabine appeared to be eliminated in a biphasic manner, with faster kinetics during the first 4–6 h followed by slower kinetics up to 24 h. Supplementary Table 4 summarizes the main PK parameters. Mean *C*_max_ and AUC_0−∞_ values on day 5 exceeded those on day 1 by 581–414 and 2691.18–2566.29, respectively. The steady state was reached after consecutive 5-day intravenous infusion. The plateau plasma concentration was 102.14±14.53 ng/ml.

This study indicated that single-agent clofarabine was active and well tolerated in heavily pretreated pediatric patients with R/R ALL in China. The ORR in this study was 44.2% (2 CRs, 17 CRis), which was quite encouraging considering the heavily pretreated population enrolled. The activities of clofarabine compared favorably with prior studies evaluating single-agent clofarabine. Hagop *et al.*^7^ observed one CR and nine CRps (CR with incomplete platelet recovery) in 12 ALL patients who received clofarabine monotherapy, while Jeha *et al.*^4^ reported a 19.7% CR+CRp and a 30% ORR in 61 pediatric ALL patients. An European study enrolled 53 pediatric patients with pretreated R/R ALL, in which 28% patients achieved CR or CRp.^5^ Our study had a numerically higher ORR (44.2%, 2 CRs plus 17 CRis) than that reported in previous studies. The results should be considered in the context that new evaluation criteria were introduced, which use CRi instead of CRp and partial response (complete disappearance of circulating blasts, ⩾5% and <25% bone marrow blasts and normal progenitor cells or <5% bone marrow blasts that did not qualify for CR or CRp) in previous criteria. Moreover, this study was a single-arm design with limited sample size, which also contributed to the results. We also see clinical benefit of clofarabine in terms of duration of remission. The median duration of remission in this study was 14.6 weeks, which allowed patients with suitable donors to proceed to hematopoietic stem cell transplantation and prolonged the survival.

A favorable toxicity profile was also seen. Most toxicity was tolerable and reversible. Grade 3 or 4 hepatic toxicities were recorded in 20.5% patients (9/44), compared with 15–46% in a previous study.^4^ Myelosuppression-associated complications were ~88.6–93.2%, which were slightly higher than the data reported (31–81%) in prior study.^10^ These findings suggest that close monitoring is needed on liver function and blood count during clofarabine administration. The toxicity profile was consistent with the prior studies of clofarabine monotherapy, and no unexpected toxicities or treatment-related death was reported in the present study.

Population pharmacokinetics study of clofarabine demonstrated that the systemic clearance and volume of distribution at steady state were 28.9 L/h/m^2^ and 170 L/m^2^, respectively, with an estimated terminal half-life of 6.2 h in Western children.^11^ In our study, the above-mentioned parameters were 20.42 L/h/m^2^, 186.66 L/m^2^ and 6.43 h, respectively. The heterogeneity of clofarabine among the individuals and limited sample size contributed to the results.

In summary, the results from this study revealed that clofarabine as a single agent is active and well tolerated in heavily pretreated Chinese pediatric patients with R/R ALL. Further studies are needed through other hematologic and solid tumors, adults and in combination strategies.

## Figures and Tables

**Figure 1:**
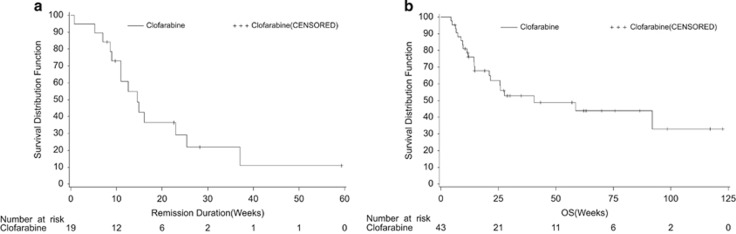
Kaplan−Meier curve for duration of remission. (**a**) Overall duration of remission and **(b)** overall survival.

**Table 1:** Patient characteristics (*N*=43)

*Characteristics*	*No. of patients*	*%*
*Age (years)*		
Median (range)	13 (5–22)	
		
*Gender*		
Male	33	76.7
Female	10	23.3
		
*Immunophenotype*		
B precursor	33	76.7
T cell	9	20.9
Other or unknown	1	2.4
		
*Previous chemotherapy*		
2	8	18.6
3	29	67.4
4	3	7
5	3	7
		
*No. of prior transplantations*		
1	4	9.3
		
*Response to last chemotherapy*^a^		
CR	21	48.8
PR	4	9.3
NR	17	39.5
Unknown	1	2.4
		
*Course of disease (week)*		
Median (range)	99.0 (11.3–460.1)	

a Abbreviations: CR, complete remission; NR, no response; PR, partial response.
