# Supplementary material for: Efficacy, safety and pharmacokinetics of clofarabine in Chinese pediatric patients with refractory or relapsed acute lymphoblastic leukemia: a phase II, multi-center study
Source: Blood Cancer J. 2016 Feb 26;6(2):e400–. doi: 10.1038/bcj.2016.8 (PMC4771971; doi:10.1038/bcj.2016.8)
Supplement: Supplementary Information [file bcj20168x1.docx]

**SUPPLEMENTARY INFORMATION**

**Efficacy, safety and pharmacokinetic of clofarabine in Chinese pediatric patients with refractory or relapsed acute lymphoblastic leukemia: a phase II, multi-center study**

Aidong LU et al.

**Supplemental Methods**

Patient eligibility

Eligible patients were pediatric patients (younger than 21 years old) with second or subsequent refractory or relapsed ALL confirmed by histology who had received at least two drugs treatment. Other eligibility criteria included the followings: 1) no prior chemotherapy within 2 weeks before entry and resolution of toxic effects from prior therapy; 2) normal cardiac function, adequate hepatic function [total bilirubin ≤1.5× upper limit of normal (ULN), aspartate aminotransferase (AST) alanine aminotransferase (ALT) ≤ 3ULN] and renal function (serum creatinine ≤ 2 ULN); 3) Eastern Cooperative Oncology Group (ECOG) performance status 0-2 and life expectancy more than 3 months. The study was amended to exclude patients with AEs not recovered from prior therapy; within 3 months from allogeneic or autologous stem cell transplantation; with central nervous involvement or uncontrolled infection. Patients who used clofarabine before or allergic to fludarabine or cladribine were also ineligible.

**Response criteria for blood and bone marrow**

1 Complete response (CR)

- No circulating blasts or extramedullary disease

No lymphaadenopathy, splenomegaly, skin/gum inflitration/testicular mass/CNS involvement

- Trilineage hematopoiesis (TLH) and ＜5% blasts
- ANC ＞ 1000/microL
- Platelets ＞ 100000/microL
- No recurrence for 4 weeks

2 Complete response with incomplete recovery of counts (CRi)

- Recovery of platelets but ＜ 100000 or ANC is ＜ 1000/microL

3 Overall reponse rate (ORR=CR+CRi)

4 Refractory disease

- Failure to achieve CR at the end of induction

5 Progressive disease

- Increase of at least 25% in the absolute number of circulating or bone marrow blasts or development of extramedullary disease

6 Relapsed disease

- Reappearance of blasts in the blood or bone marrow (＞5%) or in any extramedullary site after a CR

**Pharmacokinetic analyses**

Four patients were signed written informed consent to blood draws for pharmacokinetic determination. The blood (3 ml) and urine samples were respectively collected at predetermined time points before or after the first and fifth dosing. The plasma was separated after centrifugation and then stored at -80°C until analysis. A high-performance liquid chromatographic/tandem mass spectrometry (HPLC-MS/ MS) method has been developed and validated for the determination of clofarabine in plasma.

Supplementary Table 1 **Time point for plasma collection***

|  | **0 h** | **1 h** | **2 h** | **3 h** | **4 h** | **5 h** | **8 h** | **12 h** | **18 h** | **24 h** |
| --- | --- | --- | --- | --- | --- | --- | --- | --- | --- | --- |
| **Day 1** | **●** | **●** | **●** | **●** | **●** | **●** | **●** | **●** | **●** | **●** |
| **Day 2** | **●** |  |  |  |  |  |  |  |  |  |
| **Day 3** | **●** |  |  |  |  |  |  |  |  |  |
| **Day 4** | **●** |  |  |  |  |  |  |  |  |  |
| **Day 5** | **●** | **●** | **●** | **●** | **●** | **●** | **●** | **●** | **●** | **●** |

* It is calculated from the time of administration of clofarabine, for example, 0 hour means before administration of clofarabine.

**Detailed SAE**

Four patients (9.1%) experienced severe AEs (SAE). Two of them had elevated transaminases related to study drug and recovered after treatment for liver protection. One patient who had undergone HSCT before clofarabine treatment had elevated transaminase and severe rash, and recovered eventually, which was assessed to possibly relate to GVHD, not to study drug. One patient died of respiratory failure, the patient had a fungal pneumonia and severe myelosuppression when enrollment, the infection got worse 3 days after clofarabine administration, and the study drug was stopped immediately, the patient died on the fourth day of study.

**Supplementary Figure Legend**

**Supplementary Figure 1.** Plasma pharmacokinetics after first infusion of clofarabine
