# Supplementary figures and images for: Efficacy, safety and pharmacokinetics of clofarabine in Chinese pediatric patients with refractory or relapsed acute lymphoblastic leukemia: a phase II, multi-center study
Source: Blood Cancer J. 2016 Feb 26;6(2):e400–. doi: 10.1038/bcj.2016.8 (PMC4771971; doi:10.1038/bcj.2016.8)

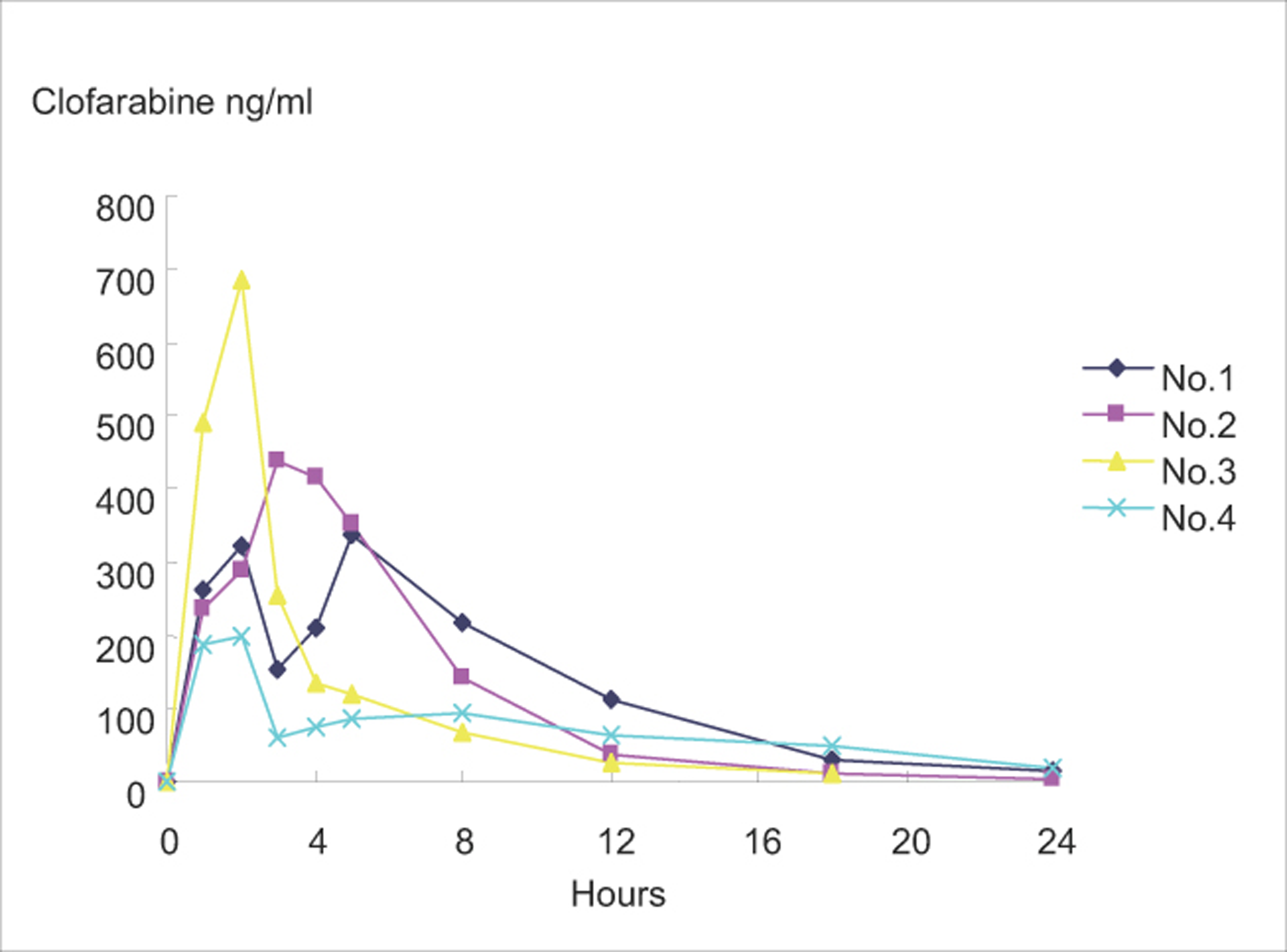

Supplement: Supplementary Figure 1 [file bcj20168x2.tif]
